# Understanding social attachment as a window into the neural basis of prosocial behavior

**DOI:** 10.3389/fneur.2023.1247480

**Published:** 2023-10-05

**Authors:** Kristen M. Berendzen

**Affiliations:** ^1^Department of Psychiatry and Biological Sciences, University of California, San Francisco, San Francisco, CA, United States; ^2^Weill Institute for Neurosciences, University of California, San Francisco, San Francisco, CA, United States

**Keywords:** attachment, prosocial behavior, values, comparative neurobiology, pair bonds, oxytocin

## Abstract

The representation and demonstration of human values are intimately tied to our status as a social species. Humans are relatively unique in our ability to form enduring social attachments, characterized by the development of a selective bond that persists over time. Such relationships include the bonds between parents and offspring, pair bonds between partners and other affiliative contacts, in addition to group relationships to which we may form direct and symbolic affiliations. Many of the cognitive and behavioral processes thought to be linked to our capacity for social attachment—including consolation, empathy, and social motivation, and the implicated neural circuits mediating these constructs, are shared with those thought to be important for the representation of prosocial values. This perspective piece will examine the hypothesis that our ability to form such long-term bonds may play an essential role in the construction of human values and ethical systems, and that components of prosocial behaviors are shared across species. Humans are one of a few species that form such long-term and exclusive attachments and our understanding of the neurobiology underlying attachment behavior has been advanced by studying behavior in non-human animals. The overlap in behavioral and affective constructs underlying attachment behavior and value representation is discussed, followed by evidence from other species that demonstrate attachment behavior that supports the overlapping neurobiological basis for social bonds and prosocial behavior. The understanding of attachment biology has broad implications for human health as well as for understanding the basis for and variations in prosocial behavior.

## Introduction

Social attachments are essential components of human social behavior, forming the basis for relationships between parents and offspring, romantic partners, friends, and even the bonds felt toward ideological or cultural groups. Attachment behavior is often defined by the selective, enduring bonds formed between offspring and a parent or caregiver as well as between unrelated partners or peers in adolescence and adulthood (1–3). A core feature of attachment theory is the existence of a caregiving relationship, typically from adult to child, that is fundamental to guide the development of prosocial behaviors such as empathy, cooperation, reciprocity, and consolation (4–7). Across species, prosocial behaviors are defined by the voluntary actions intended to benefit other individuals (8). In humans, such prosocial behaviors and attitudes are commonly integrated into culturally shaped individual values, defined as the motivations, beliefs, and goals that guide behavior (9). The innate construction of an attachment framework in early development, shaped by learned cultural attitudes and subsequent social experiences, may provide a primary scaffold upon which moral principles and value systems are built.

Early attachment experiences, as formalized by Bowlby and Ainsworth (1, 2, 10), have primarily been described in the relationships between parents and offspring, which are thought to have longstanding effects on subsequent close relationships as well as on broader social development (11–13). Subsequent attachments between mates are typically organized around the formation and maintenance of pair bonds (11, 14). These bonds are also associated with physiological distress upon separation from the pair-mate, and reduced anxiety with reunion (11, 15, 16). Regardless of social, marriage, or mating systems across cultures, pair-bonds are a ubiquitous feature of human relationships (17, 18). Humans are not unique in their ability to form such bonds, but are one of the 3–5% of mammals that form sustained, selective affiliations (19–21). Similarly, prosocial behaviors are identified across species, and in non-human animals may include grooming, support and protection, and food sharing. The neurobiology underlying long-term bonds in both humans and non-human species has been a focus of intense interest and study over the past several decades and is thought to be conserved across species that display attachment behaviors (22–24). The high level of conservation hints at the centrality of these behaviors across phylogeny and indicates that considering the biology of model organisms alongside theories of human social organization may provide powerful entry points into understanding prosocial behavior.

While prior work has explored the link between attachment behavior and the development of prosocial values (4, 6, 25), the perspective that follows suggests that the antecedents to prosocial and ethical behavior exist across species that form long term attachments. This is not to suggest that there is equivalence in the affective and behavioral states experienced, but that there are powerful precursors to prosocial processes present in non-human species. The great apes, some species of monkeys, and birds, among other species, demonstrate behavioral and affective features that approximate those of human social emotions (26–29). Here, I examine two components of prosocial values, social openness (defined as the tendency toward social contact and approach over avoidance or fear) and empathy as examples of constructs that are strongly linked to attachment behavior. I then examine the evidence for conserved underlying neurobiology mediating attachment behavior and prosocial values. Disruptions to these processes play a role in neuropsychiatric diseases that preferentially impair attachment behavior. An understanding of the overlapping neurobiology may have relevance not only to cognitive health but to social health more broadly.

## Integrating social attachment and prosocial behavior

The relationship between social attachment and prosocial behavior has been examined previously in the psychological literature (4, 23, 30). Shaver et al. in their Handbook of Attachment put forward that attachment theory, which describes a framework for social-emotional behavior primarily through development (2, 12) is, fundamentally, “a theory of prosocial behavior” (13). Interactions with attachment figures, commonly parental figures, through development shape the mental representations of others. When positive, these attachment relationships provide an enduring sense of safety and security, and the ability to recognize and regulate emotions (7, 13). Studies in adolescents find that secure attachment to parents contributes positively to compassionate, empathic responses to people in need (31, 32). Studies that have directly examined the relationship between the development of social values and adult attachment find that more secure attachments in adulthood are associated with increased prosociality, as measured by social value orientation (the balance of an individual's preference to allocate resources to the self or to others) (33, 34). Similarly, attachment quality and style with a primary caregiver are associated with the degree of altruistic helping seen in adults (35). Attachment style is correlated with the degree of exploration, curiosity, empathy, as well as fear of strangers and openness to others exhibited by adults (5, 36).

Comparative studies across species allow us to observe the “primitive” underpinnings of moral behavior in animals, while also allowing for experimental manipulation of neurobiological mechanisms involved in the representation of constructs such as empathy, fairness, reciprocity, social reward, and social openness (37, 38). A wealth of data from other species suggests not only that there is a neural substrate for attachment and prosocial behavior, but that it also developed by evolutionary selection (19, 39). Across species, social attachments have been defined by similar patterns of behavior, including mate (or pair) bonding, biparental care, and peer affiliation (36, 40). Adult pair bonds are characterized by long-term, preferential mating between two individuals and the active rejection of novel potential mates (14, 17, 41). In non-human primates, prosocial behaviors are present, including reciprocity, mutual assistance, retributive justice, reconciliation, consolation and openness to social engagement and sustained contact (42). Among these, social approach vs. avoidance and empathy and consolation are thought to be shared across species, including in rodents (30, 42, 43).

Our ability to engage in cooperation, sharing, and helping, all key components of prosocial behavior, depends upon a tendency toward social approach as opposed to social threat and fear. However, equally essential to successful social navigation is the selective engagement of such prosocial behaviors within a social network stratified by the strength of attachment relationships (44). The biological function of an innate attachment system is thought to serve to obtain or maintain proximity to significant others and caregivers in times of need or in the presence of threats, and thus to regulate support seeking behavior (4). Across species, social affiliation requires reduced physical distance and reduced threat or fear responses with close contact. In non-human animals, attachment is often measured as selective proximity-seeking and maintenance between individuals. This has been operationalized, for example, in partner-preference tests used to assess pair bonding in prairie voles, socially monogamous species that form long-term attachments (45, 46). The maintenance of proximity by two animals has been conceptualized as a cooperative behavior, one that facilitates and comes to define the pair bond (47). Social engagement and broader prosocial behaviors, including resource sharing, care-taking, and consolation, require a perception of safety, the capacity for which may be established by the nature of early attachment experiences (30).

Early attachment relationships may also shape our capacity for empathy, an essential component of prosocial values. Empathy comprises both the sharing of emotions between individuals and the adopting of another's point of view. The communication of emotional states (“emotion contagion”) as well as consolation behavior and reconciliation are components of empathy that can be examined in non-human animals. The latter two may reflect cognitive processes required for perspective taking (26). Highly social animals, such as humans, apes, corvids, and elephants, show both aspects of empathic response (26, 28, 29, 48). Consolation and reconciliation behavior have been well-characterized in chimpanzees (49, 50). Rhesus monkeys will refuse to pull a chain that delivers food to themselves if doing so shocks a companion (51, 52). Emotion contagion is likely to be present even in rodent species (53, 54). Church (53) found that rats that press a lever to obtain food, stop lever-pressing if that action is paired with delivery of a shock to a neighboring rat. The communication of emotional state is well-described across species and may have a basis in synchronized neural activity between interacting individuals. In a recent study, pairs of socially interacting mice exhibited interbrain correlations of neural activity in prefrontal cortex (PFC) that predicted future social interactions (55). While consolation behavior has not been described in commonly studied rodent species like mice and rats, prairie voles do exhibit consolation behavior characterized by allogrooming of a stressed companion (56). Such findings of shared emotional states and consolation behaviors have supported the view that non-human animals exhibit primitive, but likely neurobiologically conserved, forms of empathy (43, 57).

## Overlapping neurobiology of attachment and prosocial behavior

Compared to other species, human and non-human primate maternal and pair-bonding behaviors are more complex and flexible and are likely shaped to a greater degree by early experience. However, the underlying circuitry mediating such bonding behaviors is likely conserved across species. Activity in regions including the amygdala, ventrotegmental area (VTA), hippocampus, anterior cingulate cortex (ACC), insula, and temporal cortex has been implicated in attachment behavior in humans and other species (23). The ACC, in particular, processes information integrating social affective and representational processes (58). The insula, particularly the anterior insula, is thought to encode interoception, as well as affective states associated with physiological processes across species (59). Studies of attachment-related neural responses in humans have commonly examined parents' neural responses to their own infant vs. an unfamiliar infant. Increased connectivity between the ACC and anterior insula is found when parents view their own infant– supporting a hypothesis that synchronized activity across these regions consolidates attachment representations (60). The ACC and anterior insula are also highly implicated in empathic responses in humans, particularly in studies examining empathic pain (61). In comparison to neutral situations, painful conditions elicit significant activation in these regions. The ACC in rats may encode a primitive of fear or pain contagion as neurons in this area respond to both experienced pain and the pain of others (62), and insular cortex in rats mediates age-dependent approach vs avoidance of stressed conspecifics (63). While these regions do not selectively encode attachment and support multiple affective processes associated with social and non-social contexts, research across species has repeatedly implicated these regions in the formation and maintenance of long-term bonds (23, 24).

Work across species has shown that specific neuroendocrine mediators, in particular oxytocin, may act on similar neural circuitry to that described above to mediate many of the correlates to prosocial behavior (64, 65). Oxytocin has been linked to a host of prosocial processes and particularly to attachment behaviors across species. In humans, the oxytocin receptor (OTR) has been associated with empathy, emotion recognition, and socioemotional engagement (66–68). Oxytocin has pleiotropic actions in the brain, but is thought to mediate threat states, somatic and visceral encoding, including pain responses, as well as cognitive processes related to learning and memory and reward as they apply to social behavior (69). The effects of peripheral administration of oxytocin have been described across species with regards to prosocial and cooperative behaviors. In primates as well as monkeys, oxytocin administration facilitates cooperation and pair bonding (70, 71). Marmosets given intranasal oxytocin initiated more bouts of huddling than non-treated animals, and administration of antagonists to OTR eliminated food-sharing between partners (71). In prairie voles, where the oxytocinergic system has perhaps been most extensively studied for its role in attachment, OTR is highly expressed relative to non-monogamous species in the ACC, PLC, anterior insula, and NAc (72, 73). OTR antagonism in the ACC in voles specifically abolished consolation responses toward cagemates that experienced an unobserved stressor (56). In mice, intranasal oxytocin enhances observational fear as well as neural activity within the ACC (74). While it has become increasingly evident that the role of oxytocin in regulating social behaviors is complex and highly context- and stimulus-dependent (75–77), it remains a candidate for coordinating and organizing the underlying components of prosocial behaviors discussed above.

## Attachment behavior influences health across the lifespan

It is clear that attachment behavior has profound implications for human health. The development of close relationships early in life is essential for defining one's identity and group affiliations (78, 79) and in surviving to mate and raise offspring. Further, the formation and maintenance of long-term bonds has profound effects on physical and mental health throughout the lifespan (80–82). Intact, close social relationships consistently confer a benefit on diverse health outcomes, while the loss of close relationships and isolation have profound detrimental effects on human health. For example, stronger social relationships, measured by relationship quality, decrease the risk for all-cause morality by 50% (82), similar in effect size to interventions related to diet and physical activity (83). Conversely, decreased social interaction is significantly associated with incident dementia, with a relative risk similar to that of other established risk factors, such as low educational attainment, inactivity, and late-life depression (84, 85). Data across numerous studies reveal a clear effect of disrupted attachment relationships on all-cause mortality, cardiovascular health, metabolic function, and dementia (86–91).

Interestingly, the same circuits and brain networks implicated in attachment are those commonly disrupted across neuropsychiatric diseases that affect prosocial behaviors, such as behavioral variant frontotemporal dementia (bvFTD) (92–95). bvFTD is characterized by a loss of empathy and often impulsive, disregard for social norms (96), which fundamentally disrupt relationships with attachment figures. These social and emotional deficits correlate with significant degeneration in ACC and orbital frontoinsula (97, 98). The overlap between attachment neurobiology and the circuitry implicated in prosocial deficits in disease highlights the conservation of the underlying processes and their relevance to human health. In this issue Raya et al. propose that the rigidity and perseveration exhibited by patients with FTD reflects a decrease in openness that is linked to atrophy of dlPFC and ACC (99, 100). The deficits in empathy may also involve altered activity in the right anterior temporal lobe and medial frontal regions in FTD patients (101). Such disruptions to the neural circuitry of attachment have profound implications for patient quality of life as well. In dementia patients, and particularly those with bvFTD, decreases in empathy are associated with relationship dissolution and infidelity (102). Further, a rich body of literature has focused on the interactions between caregivers, who are often family or spouses and other attachment figures, and dementia patients and the impact on caregiver wellbeing and health (103–105). While caregivers of those with chronic conditions have been noted to exhibit increased empathy and prosocial behaviors in some studies (106), the ability to maintain attachments with the care recipient may be impacted by conditions like bvFTD with subsequent adverse effects on health outcomes for both the patient and caregiver.

One can also turn to attachment neurobiology to examine deviations from prosocial attitudes that support the values described above of empathy, compassion, reciprocity, etc. Our tendency for inter-group violence, prejudice, and bias may reflect another side of the same attachment biology (107). The development of the circuitry underlying attachment early in life drives the display of culturally normative pro-social values later in life, but may also facilitate tendencies toward out-group bias and persecution. Severe disruptions to attachment development result in profound adult social deficits (3, 108). Neglect from early attachment figures may lead to impaired bond formation later in life, as well as impulsive behaviors including violence (5). Even with typical attachment development, the formation of culturally-derived value systems and intragroup attachment is intricately tied to the neurobiology of human ethnocentrism—the tendency to judge other cultures based on standards of one's own culture (109). Such group-directed prosocial processes may simultaneously promote intergroup “antisocial” tendencies. These processes have relevance in considering care and treatment for dementia patients at both an individual and societal level. It is well documented that conditions like dementia and other neuropsychiatric diseases that may impair attachment behavior continue to be stigmatized (110, 111). This is particularly so for minoritized populations with neurodegenerative conditions, leading to decreased access to and quality of care (112, 113). Deficits in attachment and prosocial behavior that occur in conditions like FTD may further exacerbate stigmatization and ethnocentrism already demonstrated toward patient populations. Understanding these innate tendencies as reactions of the same neural system will help to elucidate both our profound capacity for prosocial and altruistic action as well as the selective withdrawal of such compassionate behaviors toward those of other groups.

The neuroendocrine mechanisms described above may provide insight into the seemingly dichotomous roles of the attachment system in mediating value-based behaviors. One prominent theory regarding oxytocin's effects on behavior suggests that while oxytocin acts to motivate in-group preference and cooperation, it simultaneously promotes out-group “derogation” (77, 109, 114). Several studies in humans have shown that peripheral administration of oxytocin is associated with increased in-group bias and that oxytocin may facilitate the emergence of intergroup conflict and violence (114, 115). In the context of attachment more specifically, the formation of a preference for a partner across species is also accompanied by rejection, often aggressively, of a novel mate (14, 40). Thus, understanding the antisocial correlates of attachment neurobiology may be key to examining the etiology of prejudice, xenophobia, and intergroup violence.

## Conclusion

Our unique ability to display selective affiliation not only with other members of our species throughout life but with social constructs such as nationality, religion, and social identity forms the basis for societal values and prosocial ethics. Early relational experiences direct the development and patterning of prosocial motivations and behaviors and have profound effects on brain health later in life (3, 5). The potential for attachment behaviors to serve as proxies in other species for components of value-based behaviors may allow us to examine, manipulate, and causally interpret such behaviors in a way that has previously not been possible in the study of human values. Comparative work on the neurobiology of attachment offers entry points into the circuitry underlying value evolution, formation, and structure as well as the mechanisms underlying disruptions to value systems in disease and common variations of human social behavior. Leveraging such understanding may allow for interventions that facilitate attachment to diverse groups and ideologies, consequently expanding prosocial responses to broader populations while reducing intergroup bias (116). Interventions such as attachment-based family therapy or school-based holistic intervention programs that are focused on early-life interactions between family members and peers have proven beneficial in promoting prosocial behaviors in children and adolescents (117–119). Adapting such programs to other stages of the lifespan may lessen neuropsychiatric symptom burden in certain populations, reduce caregiver burnout, and enhance overall quality of life for both patients and care providers (105, 120–122). Thus, a deeper neurobiological understanding of prosocial thinking and the early attachment experiences that shape it may facilitate our progression toward a more inclusive and global moral position.

## Data availability statement

The original contributions presented in the study are included in the article/supplementary material, further inquiries can be directed to the corresponding author.

## Author contributions

KB conceptualized, planned, and wrote the manuscript.
